# Evaluation of a Commercial Rapid Molecular Point-of-Care Assay for Differential Diagnosis Between SARS-CoV-2 and Flu A/B Infections in a Pediatric Setting

**DOI:** 10.3390/v16101638

**Published:** 2024-10-20

**Authors:** Paolo Bottino, Costanza Massarino, Christian Leli, Elisabetta Scomparin, Cristina Bara, Franca Gotta, Elisa Cornaglia, Enrico Felici, Michela Gentile, Sara Ranzan, Alessia Francese, Francesca Ugo, Serena Penpa, Annalisa Roveta, Antonio Maconi, Andrea Rocchetti

**Affiliations:** 1 Microbiology and Virology Laboratory, University Hospital “SS Antonio e Biagio e C. Arrigo”, Via Venezia 16, 15121 Alessandria, Italy; christian.leli@ospedale.al.it (C.L.); escomparin@ospedale.al.it (E.S.); cbara@ospedale.al.it (C.B.); fgotta@ospedale.al.it (F.G.); ecornaglia@ospedale.al.it (E.C.); arocchetti@ospedale.al.it (A.R.); 2 Research and Innovation Department (DAIRI), University Hospital “SS. Antonio e Biagio e C. Arrigo”, Via Venezia 16, 15121 Alessandria, Italy; costanza.massarino@ospedale.al.it (C.M.); alessia.francese@ospedale.al.it (A.F.); fugo@ospedale.al.it (F.U.); serena.penpa@ospedale.al.it (S.P.); aroveta@ospedale.al.it (A.R.); amaconi@ospedale.al.it (A.M.); 3 Pediatric and Pediatric Emergency Unit, University Hospital “SS. Antonio e Biagio e C. Arrigo”, Via Venezia 16, 15121 Alessandria, Italy; enrico.felici@ospedale.al.it (E.F.); migentile@ospedale.al.it (M.G.); 4 Department of Science and Technological Innovation (DISIT), University of Eastern Piedmont, Viale Teresa Michel 12 11, 15121 Alessandria, Italy; sararanzan12@gmail.com

**Keywords:** point-of-care testing, SARS-CoV-2, influenza A/B, rapid molecular assay, ID NOW, pediatric respiratory infections

## Abstract

Given the ongoing COVID-19 pandemic, there is a need to identify SARS-CoV-2 and to differentiate it from other respiratory viral infections, especially influenza A and B, in various critical settings. Since their introduction, the use of rapid antigen tests has spread worldwide, but there is variability in their diagnostic accuracy. In the present study, we evaluated the clinical performance of the ID NOW™ COVID-19 2.0, a molecular point-of-care test (POCT) based on enzymatic isothermal amplification for the differential diagnosis of SARS-CoV-2 and influenza A/B in a pediatric emergency setting. A cohort of pediatric patients admitted between December 2022 and February 2023 were simultaneously tested with the POCT and standard laboratory molecular assay. Our findings showed high negative agreement of the POCT assay across the different age groups for SARS-CoV-2, influenza A, and influenza B (more than 98.0%), while its positive agreement varied significantly for the abovementioned viral species from 50.0% to 100%. These results highlight the potential of the ID NOW™ COVID-19 2.0 POCT assay as a reliable and rapid tool for excluding SARS-CoV-2 and influenza A/B infections in symptomatic pediatric patients, although its variable positive agreement suggests a need for confirmatory RT-qPCR testing in certain clinical and epidemiological settings in order to ensure accurate diagnosis and appropriate patient management.

## 1. Introduction

Lower respiratory tract infections (LTRIs) represent one of the leading causes of deaths among children under 5 years and adults worldwide [1,2]. In 2019, the first cases of a respiratory infection caused by a previously unknown coronavirus named severe acute respiratory syndrome coronavirus 2 (SARS-CoV-2) occurred in China, spreading globally and causing a pandemic known as coronavirus disease 2019 (COVID-19) [3]. By January 2024, the number of cases reported to the World Health Organization (WHO) had surpassed 700 million [4]. SARS-CoV-2 infections generally present with initial respiratory symptoms that may evolve towards pneumonia or acute respiratory distress syndrome (ARDS). Severe clinical complications have occurred frequently in adults, while symptoms are often absent or mild in children [3].

Besides SARS-CoV-2, influenza viruses are also responsible for respiratory illnesses, especially types A and B, which represent the main culprits for infection peaks during the winter season [5]. Annually, influenza has a huge impact worldwide, causing thousands of deaths each year due to its ability to evolve through mutations and re-assortment of viral genomes [6]. Early on during the COVID-19 pandemic, influenza circulation collapsed globally due to public health measures and travel restrictions. However, there was a significant resurgence in 2022 following the removal of these restrictions [7,8]. Since SARS-CoV-2 and influenza viruses are highly transmissible and share some similarities in terms of symptom development (cough, sore throat, fever, headache, and respiratory complication), a rapid and accurate differential diagnosis is a pivotal step to prevent the further spread of the disease and provide proper treatment and patient management [9]. This challenge is exacerbated during peak respiratory virus seasons when both pathogens are circulating simultaneously, making it difficult for clinicians to distinguish between the two viral infections based solely on clinical observation [10].

Reverse transcription–quantitative polymerase chain reaction (RT-qPCR) is still considered the standard of care to identify respiratory infectious diseases; however, this method is expensive, time-consuming (3–4 h), and requires skilled technicians, thus making it difficult to process a large number of samples outside of laboratories [11,12]. The COVID-19 pandemic has led several manufacturers to develop more streamlined methods that can be performed near to the patient during the initial hospital admission. These point-of-care test (POCT) methods include immunoassays for detecting antigens and antibodies and miniaturized molecular platforms based on isothermal amplification or RT-qPCR [13]. However, they differ in terms of performance: the reported sensitivity of immunoassays varies substantially (53.8–100%) when compared to routine laboratory reference assays depending on the sample. For the molecular method, the specificity is similar across the different platforms. With increasing demand in the POCT market, several manufacturers have taken steps to develop rapid molecular assays for the simultaneous detection of influenza A virus, influenza B viruses, and SARS-CoV-2 virus [14].

The aim of this study was to compare the diagnostic accuracy of the ID NOW™ COVID-19 2.0 assay, a molecular POCT based on NEAR (nicking enzyme amplification reaction) technology, towards conventional RT-qPCR in a clinical setting of pediatric/young adult patients admitted to the emergency unit of our pediatric hospital.

## 2. Materials and Methods

**Figure 1:**
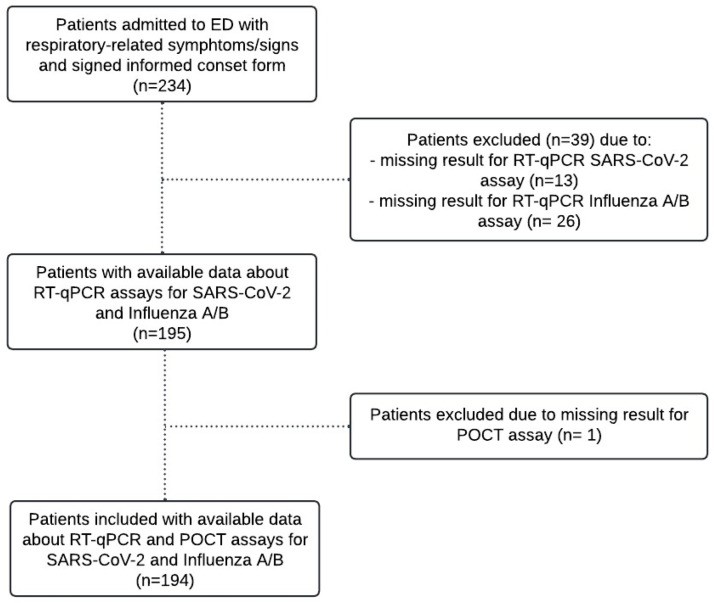
Criteria selection for enrolled patients.

Setting: In the period December 2022–February 2023, enrollment consisted of all patients aged <18 years admitted to the pediatric emergency unit of university hospital “SS. Antonio e Biagio e Cesare Arrigo” (Alessandria, Italy) for suspected respiratory infection and tested through nasopharyngeal swabs collected for clinical purposes. The trial was approved by the local ethics committee with code “ASO.Ped.20.05” and informed consent was obtained from each patient. Medical history and laboratory data were collected for each patient in addition to performing a physical examination. Where possible, we considered respiratory-related symptom similarities between the patients and those described in other studies [15,16]. Two swabs were collected: (1) a nasal swab used for the detection of SARS-CoV-2, influenza A, and influenza B using the ID NOW™ COVID-19 2.0 assay, and (2) a nasopharyngeal swab for the reference method (RT-qPCR). Of 234 patients initially enrolled, 40 were excluded due to incomplete data from either the POCT or laboratory assays. Figure 1 provides detailed information on the enrolled patients and the reasons for exclusion (39 missing RT-qPCR laboratory results and 1 with no POCT assay performed).

SARS-CoV-2/Influenza A, B Laboratory Assay (RT-qPCR): Nasopharyngeal swabs were collected in Universal Transport Medium (UTM^®^) for Viruses, Chlamydia, Mycoplasma and Ureaplasma (Copan, Brescia, Italy) and sent to the clinical microbiology laboratory for detection of SARS-CoV-2, influenza A, and influenza B genomes. Two different molecular platforms were used as the standard of care (SoC): (1) Standard M10 MDX system/SARS-CoV-2 cartridge (SD Biosensor, Suwon, Republic of Korea) for SARS-CoV-2 detection, and (2) GeneXpert System/Xpert Xpress Flu/RVS assay (Cepheid, Sunnyvale, CA, USA) for influenza A and influenza B detection. Both platforms were based on all-in-one cartridges containing nucleic acid extraction and amplification reagents for RT-qPCR, allowing to obtain results in 1 h. Detection of SARS-CoV-2 was achieved by evaluating the ORF1ab and N viral targets, while influenza A and influenza B detection was through specific genetic regions such as influenza A matrix (M), influenza A basic polymerase (PB2), influenza A acidic protein (PA), influenza B matrix (M), and influenza B non-structural protein (NS).

SARS-CoV-2/Influenza A/B POCT assay (ID NOW platform): Nasal swabs were collected in a dry tube and performed directly in the pediatric emergency department (ED) by medical staff using the ID NOW™ device and tests (Abbott, Chicago, IL, USA). Each sample was sequentially tested with the COVID-19 and then the Influenza A & B 2 assays (Abbott, USA). According to the manufacturer’s instructions, the detection of SARS-CoV-2 and influenza A/B was carried out using the same nasal swab. The assay was based on a user-friendly instrument and single cartridges for rapid isothermal amplification of SARS-CoV-2 and influenza A/B viral targets in 6 min for positive samples and less of 12 for those negative. Detection of tested viruses was achieved by evaluation of unique genetic fragments: the RdRp gene for SARS-CoV-2 and the PB2 and PA regions for influenza A and influenza B, respectively.

**Figure 2:**
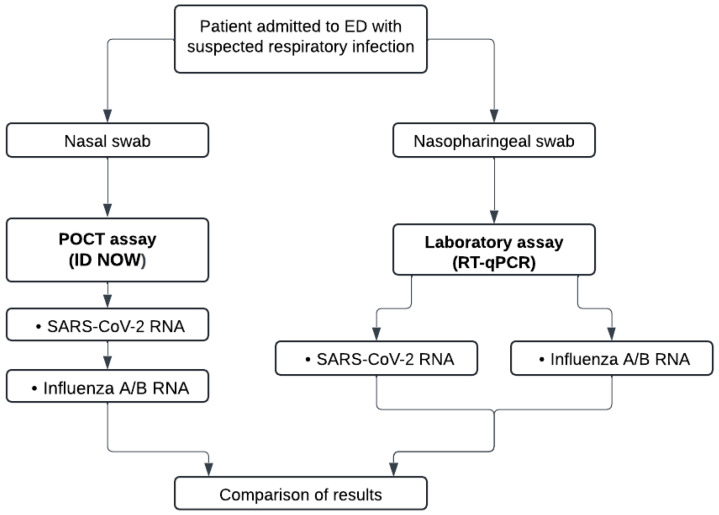
Workflows of the POCT and laboratory assays.

Figure 2 shows the workflow of the two testing strategies using either the ID NOW platform and RT-qPCR assays performed in the pediatric ED as POCT, and in the microbiology laboratory with automated platforms, respectively.

Statistical analysis: Data were collected from the laboratory information system and from ED paper medical records. All data were pseudonymized and entered into a database developed using the web-based Redcap platform. Categorical variables were described as absolute numbers and percentages, whereas continuous variables were described as medians and interquartile ranges (IQR). Positive agreement (PA), negative agreement (NA), positive predictive value (PPV), negative predictive value (NPV), positive likelihood ratio (LR+), negative likelihood ratio (LR−)—all with 95% confidence intervals (95% CI) and agreement by Cohen’s kappa between the POCT assay and laboratory RT-qPCR—were calculated as described by Hazra and Gogtay [17]. The chi-square test was used to compare the distribution of categorical variables. The results were evaluated for (1) the whole population, (2) age 0 to 3 years, and (3) age more than 3 years for SARS-CoV-2, influenza A, and influenza B. Statistical analysis was performed using Jamovi software (Version 2.3) and Medcalc (Version 22.023). The significance level was assessed at *p*-value ≤ 0.05.

## 3. Results

The study cohort consisted of 123 males (63.4%) and 71 females (36.6%), with a median age of 2.92 years (IQR: 0.89–5.83). Given the relatively young median age, the enrolled population was categorized into two age subsets: individuals aged 0 to 3 years (n = 101, 52.1%) and those over 3 years (n = 93, 47.9%). Focusing on the whole population, 7.7% were asymptomatic, while 92.3% exhibited at least one sign/symptom. The most frequent clinical presentations were body temperature ≥ 37.5 °C (81.4%), cough (67.0%), and rhinorrhea (26.8%). Other symptoms included nausea/sickness, dyspnea, diarrhea, asthenia, headache, muscle pain, and conjunctivitis. Notably, the prevalence of symptoms varied between the two age groups, with younger children (0–3 years) showing a significantly higher incidence of cough (*p*-value = 0.025), rhinorrhea (*p*-value = 0.004), and dyspnea (*p*-value = 0.045) compared to older children. Regarding biochemical parameters, the most important differences were observed for neutrophils, lymphocytes, and monocytes (*p*-value < 0.001). The clinical features of the population tested for differential diagnosis of SARS-CoV-2 and influenza A/B are presented in Table 1.

SARS-CoV-2: diagnostic performance of the ID NOW™ COVID-19 2.0 assay. Table 2 shows the diagnostic performance of the ID NOW™ COVID-19 2.0 POCT towards the RT-qPCR assay for the detection of SARS-CoV-2 across the different age subsets. For the whole population, PA and NA of the ID NOW™ COVID-19 2.0 assay were 50.0% and 98.9%, while PPV and NPV were 77.8% and 96.2%, respectively. For children aged 0–3 years, the PA was slightly higher (57.1%) with a NA of 97.9%. The PPV and NPV for this age group were 66.7% and 96.8%, respectively. In children over 3 years, the PA was 42.9% with a NA of 100%. The PPV and NPV were 100% and 95.6%, respectively. The overall accuracy of the ID NOW™ COVID-19 2.0 assay for the whole population was 95.5%, while the Cohen’s kappa coefficient was 0.58 (95% CI: 0.34–0.83; *p* < 0.0001), thus demonstrating substantial agreement.

Influenza A: diagnostic performance of the ID NOW™ COVID-19 2.0 assay: Focusing on the diagnostic performance of the ID NOW™ COVID-19 2.0 assay for the detection of influenza A in comparison with the laboratory RT-qPCR test, PA and NA were 90.0% and 100%, respectively, in the whole population; the PPV was 100%, while the NPV was 98.2%. In the 0–3 years age subset, the PA was 81.3% with a NA of 100%. The PPV and NPV were 100% and 96.6%, respectively. For children over 3 years, the ID NOW ™ COVID-19 2.0 assay achieved a PA and NA of 100%, respectively, with both PPV and NPV at 100%. The overall accuracy of the ID NOW™ COVID-19 2.0 assay for the whole population was 98.4%, while the Cohen’s kappa coefficient was 0.94 (95% CI: 0.87–1; *p* < 0.0001), thus demonstrating excellent agreement (Table 3).

Influenza B: diagnostic performance of the ID NOW™ COVID-19 2.0 assay: Table 4 shows the diagnostic performance of the ID NOW™ COVID-19 2.0 assay for influenza B detection. For the whole enrolled population, the PA was 100%, while the NA was 99.4%. The PPV the NPV were 96.4% and 100%, respectively. In the 0–3 years age group, PA, NA, PPV, and NPV were all 100%. For children older than 3 years, the PA was 100% and the NA was 98.6%. In addition, the PPV and NPV were 96.0% and 100%, respectively. The overall accuracy of the ID NOW assay in the whole enrolled cohort was 99.5%. The Cohen’s kappa coefficient was 0.98 (95% CI: 0.94–1; *p* < 0.0001), thus demonstrating excellent agreement for influenza B.

Moreover, a multiplex syndromic panel (Biofire^®^ Respiratory 2.1 panel, bioMérieux, Marcy l’Etoile, France) was performed on 33 patients, 21 of whom tested negative for both SARS-CoV-2 and influenza A/B. The most frequently detected viral pathogens were adenovirus (n = 15, 45.4%), respiratory syncytial virus (n = 11, 33.3%), human metapneumovirus (n = 8, 24.2%), human rhinovirus/enterovirus (n = 8, 24.2%), and coronavirus OC43 (n = 6, 18.2%).

## 4. Discussion

In this study, we evaluated the clinical performance of the isothermal ID NOW™ COVID-19 2.0 assay for the simultaneous detection of SARS-CoV-2 and influenza A/B viruses in comparison with the conventional RT-qPCR laboratory method used as a reference. Our results demonstrated the high performance of the POCT assay for the detection of these viruses. Rapid, accurate detection of respiratory viruses is pivotal to ensure speedy and appropriate patient management, outbreak containment, and to better understand the epidemiology of the COVID-19 pandemic [18]. The impact of the COVID-19 pandemic has reinforced the need for rapid, cost-effective, and reliable POCT devices for massive population screening and differential diagnosis at emergency departments [19]. Moreover, since SARS-CoV-2, influenza A, and influenza B viruses share similar signs and symptoms (cough, sore throat, fever, headache, and respiratory distress), an accurate differential diagnosis represents a pivotal step in order to prevent the further spread of the viral disease and to provide suitable treatment [20]. For the abovementioned reasons, several combined antigenic tests (rapid diagnostic tests or RDTs) and rapid molecular assays have been developed. RDTs are most sensitive during the first week of illness when viral loads are highest; these tests are suitable tool for use in triage with several benefits such as ease of use, cost savings, and a short turnaround time using a single device. However, RDT sensitivity is lower in asymptomatic cohorts and there is a paucity of evidence for testing in different settings [21,22]. Besides RDTs, the molecular POCT assays should offer a low-cost, highly sensitive, and specific option, especially in EDs and admission rooms. The key design features include isothermal nucleic acid amplification to avoid the need for a thermocycler, the lyophilized test reagents for improved long-term stability, and relatively simple and rapid test procedures [23,24].

In the present work, the ID NOW™ COVID-19 2.0 POCT showed an overall positive agreement of 50.0% for SARS-CoV-2 detection, with an increased value in the younger patient subset (57.1%) compared to the older patient subset (42.9%). These values were lower when compared to other studies, reporting an average sensitivity between 75% and 90% [25,26,27,28]. Nevertheless, the observed discrepancy could be attributed to differences in sample type, patient population, and testing conditions. Focusing on negative agreement, the overall value was 98.9%, with no difference in the two age subsets (0–3 years: 97.9%; >3 years: 100%), which is consistent with the findings in the studies mentioned above [25,26,27,28]. Similar studies evaluating the clinical performance of the different rapid molecular assays compared to PCR-based testing also reported high specificity but variable sensitivity, reinforcing the need for confirmatory testing in some settings [29,30].

Focusing on discrepant results between the POCT and laboratory assays, we observed two false positives with the POCT assay. Both cases involved infants less than two months old presenting with fever, cough, and dyspnea, who subsequently tested positive for respiratory syncytial virus and influenza A, respectively. Conversely, seven test results were false negative with the ID NOW™ COVID-19 2.0 assay: three tested as positive with the RT-qPCR assay, while four tested as presumptive positive, suggesting a low viral load. These patients presented with clinical evidence of respiratory infection (respiratory distress, fever, dyspnea, and cough) and were positive for viruses other than SARS-CoV-2 including influenza A (n = 3), influenza B (n = 1), and respiratory syncytial virus (n = 3) with an average Ct mean of 23.6, probably disguising the higher values for SARS-CoV-2 (>35); thus, a lower viral load is likely.

For influenza A, the ID NOW™ COVID-19 2.0 assay demonstrated a positive agreement of 90.0% and a negative agreement of 100% in the whole population, with a higher value in the older subset (100% for both positive and negative agreement), probably also due to the fewer difficulties in sample swabbing in this patients’ subset. Moreover, the high PPV and NPV observed in our study (100% and 98.2%, respectively) indicate that the ID NOW™ COVID-19 2.0 assay is highly reliable for confirming influenza A cases, thereby minimizing the likelihood of false positives. Indeed, no false positives were observed, while three patients tested false negative with the POCT assay. For two samples, the discrepancies could be explained by the high cycle threshold (Ct) values observed with the RT-qPCR assay (>37.0). The third case involved an infant aged less than one year who presented at the ED with fever. Despite a low Ct value (14.6), the discrepancy could be attributed to the young age of the patient, potentially leading to difficulties in obtaining the sample correctly.

The diagnostic performance of the ID NOW™ COVID-19 2.0 assay for influenza B showed a positive agreement of 100%, a negative agreement of 99.4%, and high PPV and NPV of 96.4% and 100%, respectively, similar to the values observed for influenza A. Only one false positive was observed: the patient, a child over three years old with an initial diagnosis of vomiting/diarrhea, exhibited no additional respiratory-like symptoms and tested negative with the laboratory RT-qPCR assay. The discrepant result could be attributed to potential contamination during either the sample collection or processing stages. These findings were consistent with the results of other studies, reporting a sensitivity and specificity for influenza A and B ranging between 90% and 100%. Noteworthy, the assays used in the present study showed variation in clinical performance depending on the timing of sample collection and the sample viral load [9,14,31].

Several studies have reported on the clinical performance of ID NOW assays for SARS-CoV-2 or flu A/B detection, showing variable sensitivity values according to epidemiological settings and symptomatic/asymptomatic population (from 60% to more than 95%), while specificity was similar in all works (>96%) [26,30,32,33,34]. In the present work, we sequentially evaluated both tests according to manufacturer’s instruction in order to provide a clinical evaluation of these tests for a timely differential diagnosis, without the need of a further swab. This latter aspect could be useful in children population to avoid a stressful procedure when patients were not fully collaboratives. Considering the epidemiological setting herein analyzed, our data provided a positive and negative agreement similar to values abovementioned, especially for influenza A and B.

The main limitations of the present study were the following: (1) the relatively low number of positive SARS-CoV-2 cases in the pediatric population, which may affect the statistical strength of the results; (2) the narrow focus on pediatric patients within a specific age range, which might not reflect the broader performance of the assay in the entire pediatric population; (3) the monocentric nature of this study, which may not represent different clinical environments or settings with variable viral loads and patient demographics features; (4) this study was performed during a limited time frame, potentially missing seasonal or epidemiological variation in virus prevalence, which could influence the diagnostic accuracy of the assay.

The rapid turnaround time of the ID NOW™ COVID-19 2.0 assay (which is able to provide results within 13 min for positive cases and 15 min for those negative) represents a significant advantage in clinical settings, particularly in the ED, where timely decision-making is critical. The fast turnaround allows for prompt isolation and treatment of infected individuals, which is pivotal in order to contain potential outbreaks. Otherwise, although more sensitive, RT-qPCR laboratory assays require more time to provide results and involve more complex laboratory infrastructure and technical expertise.

For the abovementioned reasons, the high specificity and reasonable sensitivity of the ID NOW™ COVID-19 2.0 assay make it an important tool in the diagnostic toolkit against respiratory infections, complementing but not replacing RT-qPCR laboratory platforms. However, further studies are necessary to evaluate their performance across different patient populations and clinical/epidemiological settings. Moreover, since almost all studies about this POCT are focused on its use in healthcare settings, further analysis could be aimed towards its deployment in general practitioners’ clinics. This could be particularly useful to reduce the overload of EDs during the epidemic peaks.

## 5. Conclusions

Our results demonstrate the high specificity of the ID NOW™ COVID-19 2.0 assay across different age groups for SARS-CoV-2, influenza A, and influenza B detection, making it a reliable and affordable tool for point-of-care testing in pediatric settings. The assay’s high performance in terms of specificity and positive predictive value supports its use in clinical practice, particularly for quickly ruling out these infections in symptomatic patients. However, the variability in sensitivity suggests that confirmatory testing with RT-qPCR may still be warranted in certain cases to ensure accurate diagnosis and appropriate patient management.

## Figures and Tables

**Table 1:** Clinical features of population tested for differential diagnosis of SARS-CoV-2 and influenza A/B.

Sign/Symptom	Total (n = 194)	0–3 Years (n = 101)	>3 Years (n = 93)
	N (%)	N (%)	N (%)
Asymptomatic	15 (7.7)	6 (3.1)	9 (4.6)
Symptomatic	179 (92.3)	95 (49.0)	84 (43.3)
Body temperature ≥ 37.5 °C	158 (81.4)	80 (41.2)	78 (40.2)
Cough	130 (67.0)	75 (38.7)	55 (28.4)
Rhinorrhea	52 (26.8)	36 (18.6)	16 (8.2)
Nausea/sickness	51 (26.3)	20 (10.3)	31 (16.0)
Dyspnea	48 (24.7)	31 (16.0)	17 (8.8)
Diarrhea	13 (6.7)	8 (4.1)	5 (2.6)
Asthenia	6 (3.1)	3 (1.5)	3 (1.5)
Headache	5 (2.6)	0 (0.0)	5 (2.6)
Muscle pain	5 (2.6)	1 (0.5)	4 (1.0)
Conjunctivitis	5 (2.6)	3 (1.5)	2 (1.0)
Not specified	44 (22.7)	23 (11.9)	21 (10.8)
Biochemical parameters	0–3 years (n = 78)	>3 years (n = 74)	
C-reactive protein	1.0 (0.3–3.4)	1.8 (0.4–5.6)	mg/dL
White blood cell count	10.6 (7.1–14.0)	9.7 (6.1–13.7)	×10^3^/μL
Neutrophils	50 (42–61)	73 (58–83)	%
Lymphocytes	35 (25–43)	17 (9–26)	%
Monocytes	7.4 (5.5–10.6)	4.9 (3.4–7.1)	%
Eosinophils	0.5 (0.2–1.3)	0.35 (0.1–0.7)	%
Basophils	0.4 (0.30–0.60)	0.3 (0.2–0.6)	%

**Table 2:** SARS-CoV-2: diagnostic performance of ID NOW assay.

		RT-qPCR		
		Positive (%)	Negative (%)	Total (%)
ID NOW	Whole population (n = 194)			
	Positive (%)	7 (3.6)	2 (1.0)	9 (4.6)
	Negative (%)	7 (3.6)	178 (91.8)	185 (95.4)
	Total (%)	14 (7.2)	180 (92.8)	194 (100)
	0–3 years (n = 101)			
	Positive (%)	4 (4.0)	2 (1.9)	6 (5.9)
	Negative (%)	3 (3.0)	92 (91.1)	95 (94.1)
	Total (%)	7 (7.0)	94 (93.0)	101 (100)
	>3 years (n = 93)			
	Positive (%)	3 (3.2)	0 (0.0)	3 (3.2)
	Negative (%)	4 (4.3)	86 (92.5)	90 (96.8)
	Total (%)	7.5 (7.5)	86 (92.5)	93 (100)
	Test performances			
		%	95% CI	
	Whole population (n = 194)			
	PA	50.0	23.0–77.0	
	NA	98.9	96.0–99.9	
	PPV	77.8	44.5–93.9	
	NPV	96.2	93.8–97.7	
	LR+	45.0	10.3–196.6	
	LR−	0.5	0.30–0.85	
	Accuracy	95.5%	91.4–97.9	
	0–3 years (n = 101)			
	PA	57.1	18.4–90.1	
	NA	97.9	92.5–99.7	
	PPV	66.7	30.6–90.1	
	NPV	96.8	92.9–98.6	
	LR+	26.9	5.9–122.0	
	LR−	0.4	0.2–1.0	
	Accuracy	95.0%	88.8–98.4	
	>3 years (n = 93)			
	PA	42.9	9.9–81.6	
	NA	100	95.8–100	
	PPV	100	29.2–100	
	NPV	95.6	91.9–97.6	
	LR+	NA	N/A	
	LR−	0.6	0.3–1.1	
	Accuracy	95.7	89.3–98.8	

N/A: not available.

**Table 3:** Influenza A: diagnostic performance of ID NOW assay.

		RT-qPCR		
		Positive (%)	Negative (%)	Total (%)
ID NOW	Whole population (n = 194)			
	Positive (%)	27 (13.9)	0 (0.0)	27 (13.9)
	Negative (%)	3 (1.5)	164 (84.5)	167 (86.1)
	Total (%)	30 (15.5)	164 (84.5)	194 (100)
	0–3 years (n = 101)			
	Positive (%)	13 (12.9)	0 (0.0)	13 (12.9)
	Negative (%)	3 (3.0)	85 (84.2)	88 (87.1)
	Total (%)	16 (15.8)	85 (84.2)	101 (100)
	>3 years (n = 93)			
	Positive (%)	14 (15.1)	0 (0.0)	14 (15.1)
	Negative (%)	0 (0.0)	79 (84.9)	79 (84.9)
	Total (%)	14 (15.1)	79 (84.9)	93 (100)
	Test performances			
		%	95% CI	
	Whole population (n = 194)			
	PA	90.0	73.5–97.9	
	NA	100	97.8–100	
	PPV	100	87.2–100	
	NPV	98.2	94.9–99.3	
	LR+	N/A		
	LR-	0.1	0.03–0.29	
	Accuracy	98.4	95.5–99.7	
	0–3 years (n = 101)			
	PA	81.3	54.4–96.0	
	NA	100	95.8–100	
	PPV	100	75.3–100	
	NPV	96.6	91.1–98.7	
	LR+	N/A		
	LR−	0.2	0.1–0.5	
	Accuracy	97.0	91.6–99.4	
	>3 years (n = 93)			
	PA	100	76.8–100	
	NA	100	95.4–100	
	PPV	100	76.8–100	
	NPV	100	95.4–100	
	LR+	N/A		
	LR−	0.0		
	Accuracy	100	96.1–100	

N/A: not available.

**Table 4:** Influenza B: diagnostic performance of ID NOW assay.

		RT-qPCR		
		Positive (%)	Negative (%)	Total (%)
ID NOW	Whole population (n = 194)			
	Positive (%)	27 (13.9)	1 (0.5)	28 (14.4)
	Negative (%)	0 (0.0)	166 (85.6)	166 (85.6)
	Total (%)	27 (13.9)	167 (86.1)	194 (100)
	0–3 years (n = 101)			
	Positive (%)	3 (3.0)	0 (0.0)	3 (3.0)
	Negative (%)	0 (0.0)	98 (97.0)	98 (97.0)
	Total (%)	3 (3.0)	98 (97.0)	101 (100)
	>3 years (n = 93)			
	Positive (%)	24 (25.8)	1 (1.1)	25 (26.9)
	Negative (%)	0 (0.0)	68 (73.1)	68 (73.1)
	Total (%)	24 (25.8)	69 (74.2)	93 (100)
	Test performances			
		%	95% CI	
	Whole population (n = 194)			
	PA	100	87.2–100	
	NA	99.4	96.7–100	
	PPV	96.4	79.3–99.5	
	NPV	100	97.8–100	
	LR+	167	23.7–1178.6	
	LR−	0.0		
	Accuracy	99.5	97.2–99.9	
	0–3 years (n = 101)			
	PA	100	29.2–100	
	NA	100	96.3–100	
	PPV	100	29.2–100	
	NPV	100	96.3–100	
	LR+	N/A		
	LR−	0.0		
	Accuracy	100	96.4–100	
	>3 years (n = 93)			
	PA	100	85.8–100	
	NA	98.6	92.2–100	
	PPV	96.0	77.4–99.4	
	NPV	100	94.7–100	
	LR+	69.0	9.9–482.9	
	LR−	0.00		
	Accuracy	98.9	94.1–99.9	

N/A: not available.

## Data Availability

The data contained in this manuscript are available upon request.
